# Stigma toward individuals with cannabis use disorder across age groups: associations with familiarity and sociodemographic characteristics

**DOI:** 10.1186/s12954-026-01491-1

**Published:** 2026-06-16

**Authors:** Lukas Andreas Basedow, Elisa Wandinger, Antonia Brindle, Michael Kölch, Olaf Reis

**Affiliations:** 1 Department of Clinical Psychology and Psychotherapy, Philipps-University Marburg, Marburg, Germany; 2 Department of Child and Adolescent Psychiatry and Neurology, Rostock University Medical Center, Rostock, Germany; 3 German Center for Child and Adolescent Health (DZKJ), Site Greifswald/Rostock, Rostock, Germany

**Keywords:** Adolescents, Cannabis use, Stigma, Cannabis use disorder, Attitudes, Survey research

## Abstract

**Background:**

Stigma experienced by individuals with cannabis use disorder (CUD) has been identified as a significant impediment to their engagement with treatment programs. Therefore, this study aims to investigate the effect of sociodemographic factors and familiarity with cannabis on stigmatizing attributions towards adolescent and adult case vignettes of people affected by CUD.

**Method:**

A quota sampling web survey in Germany conducted by a professional survey institute including two case vignettes of people with a CUD, applying a 2 × 2 × 2 (age of vignette* age of participant*familiarity with cannabis/CUD) within-subject design.

Sociodemographic information, questions about participants’ cannabis use experience, a measure of stigmatizing attitudes, and a short version of the attribution questionnaire.

**Results:**

A total sample of *n* = 1603 (50.1% female) participants was recruited, with *n* = 501 being adolescent participants. Subjective social standing of the whole sample was slightly elevated (Mean social rank (SD) = 5.74 (1.8)). The adolescent case with a CUD was more stigmatized compared to an adult case (F(1, 3196) = 12.77; *p* < 0.001). Individuals who were more familiar with cannabis use (F(1,3196) = 19.65; p < 0.001) and/or CUD (F(1,3196) = 6.00; *p* =0 .014) reported fewer stigmatizing attributions. This effect of familiarity was more pronounced for adolescents compared to adults.

**Conclusions:**

Adults with CUD seem to receive less stigma compared to adolescents with CUD, while stigmatizing attributions are produced less often when people have more contact with cannabis use and CUD. Future studies should aim to systematically test the influence of different characteristics of stigmatizing persons on stigma production in order to offer tailored interventions.

**Supplementary Information:**

The online version contains supplementary material available at 10.1186/s12954-026-01491-1.

## Background

Cannabis use often goes hand in hand with a number of detrimental health conditions [1, 2] including the development of a cannabis use disorder (CUD). The dysregulated use of cannabis, in form of CUD, is associated with different health problems [3, 4], is one of the most prevalent substance use disorders worldwide [5, 6], and is one of the most common reasons why adults [7] and adolescents [8] seek mental health treatment. While efficient treatment options for CUD exist [9], not all affected individuals seek treatment [10, 11] or receive the best level of available care [12, 13]. One reason for reduced treatment uptake and quality is stigma towards people with CUD [14]. Stigma is a barrier for the treatment of psychological disorders in general [15], with substance use disorders being especially affected [16]. Detrimental effects of stigmatizing attitudes on treatment have been described with regard to alcohol [17, 18] or opioid use disorder [19, 20]. For CUD, a number of studies describe stigma as a barrier for patients to disclose their cannabis use [21] and to seek CUD-specific treatments [22–26]. High levels of experienced stigmatization have been with associated a roughly 60% reduction in treatment uptake [24]. Specifically, around 10–30% of people with CUD were reluctant to seek treatment due to fear of stigmatization [22, 25, 26]. Recent global increases in cannabis use [27, 28] have been partly attributed to changes in legal status [29, 30]. At the same time, the incidence of CUD has risen, yet treatment uptake remains low, potentially due to a tendency to downplay the health risks associated with cannabis use [23]. This underscores the need for well-developed treatment pathways, including access to treatments, to address these issues effectively. Taking into account the effect of stigma on treatment uptake, one approach to increase treatment access could be stigma reduction. However, research shows that interventions focused on stigma reduction need to be specific and tailored to target populations to be effective [27, 28]. Thus, for effective measures of stigma reduction, it is essential to investigate the conditions of stigmatizing attitudes towards people affected by CUD (e.g., aiming to reduce access to resources for affected people) and stigmatizing attributions about people affected by CUD (e.g., the belief that people with CUD are lazy or incompetent).

According to the literature, a variety of conditions is associated with stigmatizing attitudes or attributions. The degree of stigmatization is influenced by the social and legal environment surrounding people who use drugs, e.g., punitive cannabis policies have been linked to higher levels of stigmatization [29, 30]. However, stigma is also related to the stigmatizing person, the persons being stigmatized, and the type of substance used by the stigmatized person [31, 32]. Relevant substance use patterns for stigmatization may regard recreational purposes, routes of administration, or the frequency of use [33]. Characteristics of the individuals producing stigmatizing attitudes, such as religious beliefs, can contribute to more pronounced stigmatization [34]. Thus, it is essential to continue stigma research with a focus on the conditions under which stigma is generated. One direction for this research line is the focus on sociodemographic factors, such as age, and their association with more or less pronounced stigmatizing attitudes. Previous studies have investigated how adults stigmatize adolescent cases of CUD [35] and have reported associations of higher age of the stigmatizer with stronger stigma [34]. The specific stigma adolescents experience from adults has not been well explored but previous research shows that attitudes of parents [36] and treatment providers [37] contribute significantly to treatment uptake. While previous research on adolescents’ stigmatizing attributions has focused on affected peers [38], adolescents can also produce stigma with regard to adults [39, 40]. This might not lead to outcomes as detrimental as the reverse case, but being stigmatized by adolescents might still impact quality of life [41, 42], family dynamics, and impair adult identity constructions [43, 44]. However, to date, no systematic research has been conducted to investigate the impact of age on the stigmatization from the perspectives of both the recipient and the producer.

As Germany has recently legalized the possession and cultivation of cannabis for recreational use [45] it provides an interesting case study for stigmatizing attitudes in the general population. Previously, lifetime prevalence of cannabis use in the German population has been estimated to be between 4 and 11% in 2024 [46, 47] providing the opportunity to conduct analyses controlling for the effects of one’s own cannabis use or familiarity with people who use cannabis in producing stigmatizing attributions. Specifically, similar to the demographic factors influencing stigmatization, familiarity with cannabis use or people who use cannabis (PWUC) might influence stigmatization [48]. A change in legal status of a substance (as with cannabis for Germany) may also influence the familiarity of the population with the substance and as such stigmatizing attributions.

Therefore, we conducted a 2 × 2 × 2 case vignette study in a representative survey of the German population with the aim of investigating how adults and adolescents differ with regard to their stigmatizing attributions about adult and adolescent cases of CUD, while controlling for familiarity with cannabis and CUD.

## Methods

### Procedure

Data collection took place from January 2024 to March 2024 using a web-based survey. Data were collected via an online survey hosted by our research team and distributed through a professional survey institute under a sample-only agreement. Participants expressing interest in the study provided informed consent and completed age- and gender-related screening items and were excluded from the survey if their demographic quota had already been filled. Age and sex quotas across five adult brackets were applied to approximate the German population distribution while individuals aged 16–18 were purposefully oversampled. Quality-control measures, including speed checks to exclude implausibly fast responses, were implemented to ensure data reliability. Participants were continuously recruited until a target quota was reached (16–18 years: n = 500; 19–29 years: n = 208; 30–39 years: n = 210; 40–49 years: n = 204; 50–59 years: n = 250; 60–75 years: n = 228; with a 50/50 gender ratio). Recruitment took longer for adolescents, so many adults had to be rejected once their quotas were met: of 9874 who consented, 1,603 completed the survey, because sampling stopped when quotas were reached. The average time spent on the survey was 5.5 min. Individual informed consent to participate in the survey was obtained from all participants prior to collecting any data. Recruitment, consent, and field procedures were approved by the local ethics committee of the University Medical Centre Rostock (#A2023-0092) and in accordance with the Declaration of Helsinki. All details are reported in line with STROBE statement [49].

Participants in the survey read two case vignettes describing a person with typical symptoms of CUD, see Supplemental Table 1 for full German and English versions of the vignettes. The two vignettes were identical except for the age of the affected person (16 vs. 50 years), their gender-neutral names (“Kaja” vs. “Conny”), and the effect of their cannabis use on their responsibilities (school vs. work).

### Materials

#### Sociodemographic questions

The survey included self-designed demographic questions, asking for the age of participants in years, their gender identity (male, female, diverse, do not want to disclose), their nationality (German, other), the size of their hometown (under 5000 inhabitants, 5000–19,999 inhabitants, 20,000–100,000 inhabitants, over 100,000 inhabitants) and their subjective social standing according to the McArthur Scale of Subjective Social Status [50].

#### Questions on familiarity with cannabis and cannabis use related problems

We asked all participants about their personal experience and familiarity with cannabis use and cannabis use related problems in the form of yes/no questions. The questions were: “Have you ever used cannabis in your life?”; “Have you used cannabis in the past 3 months?”; “Have you had a strong craving or urge to use cannabis at least once a week over the past 3 months?”; “Did somebody else express concern regarding your cannabis use in the past 3 months?”; “Do you know somebody with a cannabis use disorder?”. As these questions were analyzed on item level no reliability index was calculated.

#### General stigmatizing attitudes

*S*tigmatizing attitudes were assessed in the whole sample with a scale developed by Schomerus, Matschinger, & Angermayer [51] for alcohol. With the permission of Prof. Schomerus, we adapted this scale for CUD by replacing “alcohol” in each question with the term cannabis, see Supplemental Table 2. The scale consists of seven items describing attitudes or stereotypes towards people with a CUD. Each item is answered on a scale from 1 (“agree completely”) to 5 (“not at all”). From the total scale, three subscales can be constructed, by calculating the sum score across the included items: “Blaming” (items 1, 2, 3, Cronbach’s alpha = 0.67); “Endorsing an illness concept” (Items 4,5,6, Cronbach’s alpha = 0.58); “Trivializing the problem” (items 3,6,7, Cronbach’s alpha = 0.16). Each subscale score ranges from 3 to 15, with lower scores indicating a stronger agreement with the attitude.

#### Attribution questionnaire

After each case vignette, participants answered the short form of the Attribution Questionnaire-27 [52, 53]. While the original scale was developed for stigma regarding mental illness in general it has been successfully adapted for substance use disorders [35, 38]. The short form [54] consists of eight items describing stigmatizing attributions regarding the person from the case vignette. For each item participants rated their agreement with the statement on an eight-point scale from 1 (“not at all”) to 8 (“completely”) separately for the adolescent vignette (Cronbach’s alpha = 0.73) and the adult vignette (Cronbach’s alpha = 0.76). In line with previous studies supporting a one-dimensional structure of the AQ [55, 56] we calculated a total score (sum score of all items) with a higher score indicating stronger agreement with stigmatizing attributions.

### Statistical analysis

All data analysis was performed in Rstudio [57–61]. The sample was characterized descriptively in terms of sociodemographic variables and their cannabis use experiences. Differences in cannabis use patterns between adult and adolescent participants were calculated via chi-square tests. For the main analysis six repeated measure analyses of covariance (RM-ANCOVA) were conducted. Dependent variable was AQ-total score, with vignette type (adult vs. adolescent) as a repeated measure factor and participant age (adult vs. adolescent) as a between subject factor. For each RM-ANCOVA one of the dichotomous cannabis use questions was added as an additional between subject factor, modelled this way as a covariate. Additionally, we calculated a model investigating the effect of gender on stigmatizing attributions with the RM-ANCOVA model as described above, adding gender as the third factor instead of cannabis use familiarity. Alpha level was set to 0.05. As our analysis involved six distinct models with different covariates that explore distinct effects on our main outcome of interest, we did not correct for multiple comparisons.

## Results

### Sample description

As a result of the focused sampling procedure the final sample contained an even split regarding male and female participants (*n* = 803, 50.1% female). The mean value of subjective social standing was reported as 5.74 on a 10-point scale. Because of our sampling procedure focusing on recruiting adolescents, our sample was notably younger than the German average (36.6 years vs. 44.6 years) and has a larger proportion of participants with German citizenship (96.3% vs. 86.8%). See Table 1 for a complete overview regarding sociodemographic background.

**Table 1:** Sociodemographic background of full sample

Sociodemographic variable	Full sample (n = 1603)	German census data [62]
*Age*		
Mean (SD)	36.6 (17.5) years	44.6 years
Min—Max	16–71 years	/
Median	34 years	/
*Gender*		
Male	800 (49.9%)	49.3%
Female	803 (50.1%)	50.7%
*Subjective social standing*		
Mean social rank of self (SD)	5.74 (1.8)	/
Mean social rank of family (SD) [only asked in adolescent participants]	6.09 (1.7)	/
*Nationality*		
German	1543 (96.3%)	86.8%
Non-German	60 (3.7%)	13.2%
*Population of home town*		
Less than 5000	342 (21.3%)	13.63%
5000–19.9999	344 (21.5%)	26.55%
20.000–100.000	368 (23.0%)	27.59%
More than 100.000	549 (34.2%)	32.23%

### Cannabis use and cannabis-related beliefs

Overall, 659 (41.1%) participants had used cannabis at some point in their lives and 187 (11.6%) have used cannabis in the past three months. Additionally, 581 (36.2%) participants knew someone with CUD. Despite lifetime cannabis use (see Table 2) being more pronounced in adults (*χ*^*2*^ (1) = 18.7; *p* < 0.001), a higher proportion of adolescents reported knowing someone with CUD (*χ*^*2*^ (1) = 17.1; *p* < 0.001).

**Table 2:** Differences between adolescents and adults in cannabis use, familiarity with CUD, and stigma-related attitudes

	Adolescents (n = 501)	Adults (n = 1102)	Group difference
*Did you ever use cannabis?*			
Yes	166 (33.1%)	493 (44.7%)	*χ*^*2*^ (1) = 18.7; *p* < 0.001*
No	335 (66.9%)	609 (55.3%)	
*Have you used cannabis in the last 3 months?*			
Yes	71 (14.2%)	116 (10.5%)	*χ*^*2*^ (1) = 4.1; *p* = 0.043*
No	430 (85.8%)	986 (89.5%)	
*In the last 3 months, did you have a strong urge or craving to use cannabis?*			
Yes	45 (8.9%)	69 (6.3%)	*χ*^*2*^ (1) = 3.5; *p* = 0.063
No	456 (91.1%)	1033 (93.7%)	
*In the last 3 months, was someone worried about your cannabis use?*			
Yes	28 (5.6%)	64 (5.8%)	*χ*^*2*^ (1) = 0.003; *p* = 0.95
No	473 (94.4%)	1038 (94.2%)	
*Do you know someone with a cannabis use disorder?*			
Yes	219 (43.7%)	362 (32.9%)	*χ*^*2*^ (1) = 17.1; *p* < 0.001*
No	282 (56.3%)	740 (67.1%)	
Stigma scale – mean score “Blaming”	8.76	8.52	*t*(1068) = 1.7; *p* = 0.084
Stigma scale – mean score “Endorsing an illness concept”	8.96	8.55	*t*(1037) = 2.7; *p* = 0.007*
Stigma scale – mean score “Trivializing the problem”	8.46	8.41	*t*(1055) = 0.4; *p* = 0.698

^*^significant at the *p* < 0.05 level. The scores on the stigma scales range from 3 to 15 with lower scores indicating higher agreement with the stigmatizing attitude

### Stigmatizing attributions

**Fig. 1:**
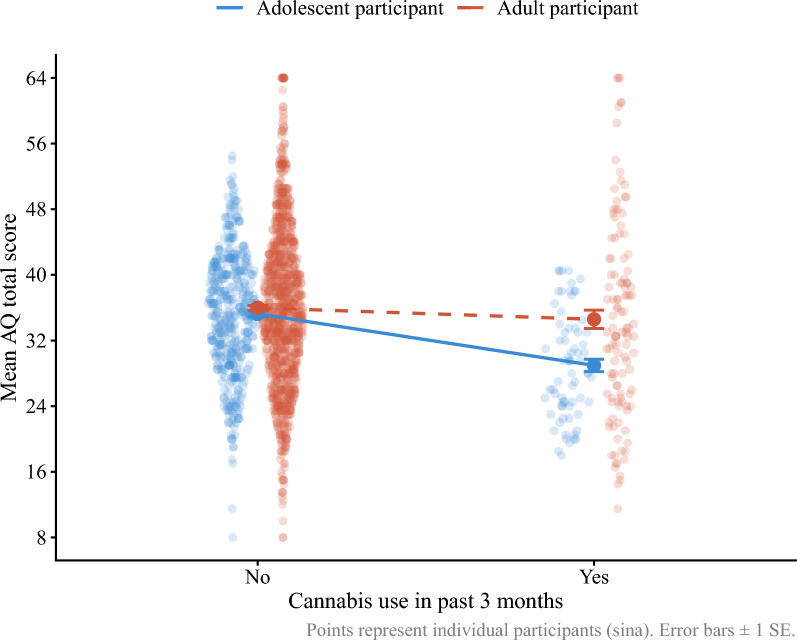
Interaction between cannabis use and participant age regarding mean score of Attribution Questionnaire

Across all six RM-ANCOVAS participants reported stronger stigmatizing attributes regarding the adolescent case vignette (all *p* < 0.001). Additionally, reporting a lifetime experience of cannabis use (F (1,3196) = 132.45; *p* < 0.001), cannabis use in the past 3 months (F (1,3196) = 37.56; *p* < 0.001), someone being worried about the participant’s cannabis use (F (1,3196) = 23.87; *p* < 0.001), and knowing someone with CUD (F (1,3196) = 13.23; *p* < 0.001) all contributed significantly to reduced stigmatizing attributes. There was also a significant interaction between cannabis use questions and participants’ age. Specifically, the effects of cannabis use in the past 3 months (F (1,3196) = 19.65; *p* < 0.001; see Fig. 1), reported craving (F (1,3196) = 4.89; *p* = 0.027), someone being worried (F(1,3196) = 21.67; *p* < 0.001), and knowing someone with CUD (F(1,3196) = 6.00; *p* = 0.014) on stigmatizing attributions, were all more pronounced in adolescents than in adults. Finally, there was no effect of gender (F(1,3196) = 1.31; *p* = 0.25) on AQ scores nor any significant interaction of vignette age (F(1,3196) = 0.004; *p* = 0.94) or participants age (F(1,3196) = 0.018; *p* = 0.89) with gender. See Supplemental Tables 3 and 4 for a complete overview.

## Discussion

This representative survey aimed to investigate differences in stigmatizing attributions between adults and adolescents, taking into account their own cannabis use. In a sample representative of the adult German population in terms of age and gender with a purposefully oversampled adolescent counterpart, we found that stigmatizing attributions were stronger when regarding an adolescent with CUD, compared to an adult. Furthermore, higher familiarity with cannabis and CUD was associated with less stigmatizing attributions, and this effect was more pronounced for adolescents compared to adults. Finally, we showed a slightly higher 3-month cannabis use prevalence in adolescents compared to adults (14.2% vs. 10%).

Our sample reported a medium level of agreement with general stigmatizing attributions with regard to CUD. These general stigmatizing attributions are in line with findings regarding alcohol use disorder [51] and other psychological disorders [63–65] in German populations, indicating a general attitude of disapproval toward people with CUD. Additionally, in line with previous literature [35], stigmatizing attributions (as measured by the AQ) seem to be more pronounced if they are aimed at adolescents with CUD. This is especially worrying, given that adolescents with substance use disorders (SUDs) are typically underrepresented in the provision of appropriate psychological and medical treatment [66]. As stigma contributes to reduced treatment uptake [67], higher levels of stigmatization might contribute to this treatment lack in a highly vulnerable population. Since we found this effect across age groups of stigmatizers, the CUD-related stigma takes also place among adolescents themselves. Thus, our results support previous studies showing that mental health stigma generally [68, 69] and CUD stigma specifically [70, 71] is commonly reported by adolescents. Stigmatization in peer-groups was found to reduce the willingness to disclose CUD to peers [72, 73] and/or to support systems [74, 75]. Generally, mental health literacy and the use of mental health services is low among adolescents [76, 77], but comparatively high levels of stigmatizing attributions might decrease this willingness even further.

While an adolescent case vignette was associated with larger stigmatizing attributions, we found that familiarity with cannabis (either through own use or knowing someone with a CUD) was consistently associated with reduced stigmatizing attributions, and this effect was more pronounced for adolescent participants. These results are in line with the so-called contact hypothesis, postulating that stigmatizing attributes can be reduced via exposure to the stigmatized behavior or persons [48, 78]. Furthermore, these results might also be of importance considering the changes in cannabis legalization happening in many countries in the world and Germany more specifically. Through a legalized pathway of access, people and especially adolescents might be exposed more to cannabis, cannabis use, and cannabis-related disorders in a process of normalization. Based on our results, this increased familiarity might be associated with reduced stigma and thus a larger treatment uptake, as it was shown for mental disorders and alcohol use disorders already [79, 80]. However, an exposure to cannabis and cannabis-related problems also provides obvious risks and thus the need for treatment for adults and adolescents might actually increase [81], offsetting the beneficial effect of reduced stigma with an increased treatment need overall. Nonetheless, our results provide support for potential prevention and stigma-reduction efforts insofar as an awareness of CUD and its acceptance as a mental disorder with effective treatment avenues might be beneficial as a stigma-reduction effort [82, 83]. Therefore, building a bridge between (former) patients with CUD of different age groups and adolescents, e.g., via social networks, can potentially reduce (self-) stigma and increase utilizations of treatment services. Based on previous research such awareness campaigns should be designed specifically for young people [84] and be culturally specific [84]. Future studies should investigate if exposing adolescents to CUD related awareness campaigns is sufficient to reduce stigma or if actual use experience is necessary.

## Limitations

First, these results are based on a cross-sectional digital survey, thus no causal claims regarding the relationship between age, cannabis use, and stigmatizing attributes can be made. Additionally, the web-based nature of the survey introduces sampling bias and risks excluding individuals without reliable internet access. However, this survey was conducted across a large and representative sample and thus highlights patterns that are likely to occur beyond the sampled participants.

Second, attributes were assessed with regard to fictional case vignettes. Even though the use of case vignettes is standard practice in stigma research, attributions regarding real people might differ.

Third, while we aimed to only investigate an age effect regarding the differences in the case vignettes, the vignettes also differed in their names and the described symptoms in line with their age (e.g., one case has problems at work because of CUD, the other case has problems at school). This variation between vignettes might also induce variation in response behavior in participants.

Furthermore, one of our key variables, familiarity with CUD, likely depends on participants’ personal understanding of what constitutes a CUD. Respondents presumably answered based on their own conception, so differences in reported familiarity between adults and adolescents may partly reflect differences in how each group perceives CUD rather than actual exposure or knowledge. Future studies might benefit from a more structured and comprehensive approach investigating lay persons’ conceptions of substance use disorders, such as CUD.

Finally, our sample seems to report a slightly higher cannabis use prevalence and higher prevalence of cannabis-related problems (see Table 2), especially among adolescents, compared to previous studies [5, 46, 47, 85]. This difference might be a result of specific biases that occur with web-based sampling [86]. Nevertheless, this higher prevalence of cannabis use should not weaken our main findings, as we specifically controlled for familiarity with cannabis use and CUD.

## Conclusion

Cannabis use is highly prevalent among German adults and adolescents, with adolescents reporting a higher 3-month prevalence than adults. Stigma with regard to CUD seems to be more pronounced for adolescent patients even among adolescents themselves, indicating the need for stigma-reduction campaigns especially focused on reducing stigma in adolescent peer-groups. Additionally, knowing people with a CUD and being exposed to cannabis use is associated with reduced stigmatizing attributions. Prevention efforts should aim to raise awareness for CUD symptoms among adolescents and aim to normalize help seeking behavior. Future research should investigate the boundary conditions under which awareness and anti-stigma campaigns successfully reduce CUD stigma.

## Supplementary Information

Additional file1 (DOCX 22 kb)

## Data Availability

The datasets used and analysed during the current study are available from the corresponding author on reasonable request.
