# Supplementary material for: Stigma toward individuals with cannabis use disorder across age groups: associations with familiarity and sociodemographic characteristics
Source: Harm Reduct J. 2026 Jun 16;23:106. doi: 10.1186/s12954-026-01491-1 (PMC13274103; doi:10.1186/s12954-026-01491-1)
Supplement: Supplementary file 1 — Additional file1 (DOCX 22 kb) [file 12954_2026_1491_MOESM1_ESM.docx]

**Stigma Toward Individuals with Cannabis Use Disorder Across Age Groups: Associations With Familiarity and Sociodemographic Characteristics**

Running head: Stigma and Cannabis Use Disorder

**Authors:**

Lukas A. Basedow^1^* lukas.basedow@uni-marburg.de (ORCID: 0000-0003-4866-8686)

Elisa Wandinger^2,3^ elisa.wandinger@med.uni-rostock.de (ORCID: 0000-0002-4367-5484)

Antonia Brindle^2,3^ antonia.brindle@med.uni-rostock.de (ORCID: 0009-0009-6005-4573)

Michael Kölch^2,3^ michael.koelch@med.uni-rostock.de (ORCID: 0000-0003-2878-5121)

Olaf Reis^2,3^ olaf.reis@med.uni-rostock.de (ORCID: 0000-0001-6480-6431)

^1^ Department of Clinical Psychology and Psychotherapy, Philipps-University Marburg, Marburg, Germany

^2^ Department of Child and Adolescent Psychiatry and Neurology, Rostock University Medical Center, Rostock, Germany

^3^ German Center for Child and Adolescent Health (DZKJ), Site Greifswald/Rostock, Rostock, Germany

*Corresponding author

**Supplemental method section**

*Supplemental Table 1.* Original case vignettes in German and an English translation.

| **German** | **English** |
| --- | --- |
| Kaja ist 16 Jahre alt, geht noch zur Schule und kifft täglich. Der Konsum ist so ausgeprägt, dass sich bei Kaja eine Cannabisabhängigkeit entwickelt hat. Sobald der Tag beginnt, denkt Kaja ans Kiffen und kann oft an nichts anderes denken. Alle von Kajas Freunden kiffen auch und unterstützen Kajas Konsum. Kaja hat schon mehrmals probiert, den Konsum zu reduzieren aber das hat nie geklappt. Durch das Kiffen hat Kaja Probleme mit der Konzentration bekommen, geht immer seltener zur Schule und schreibt immer schlechtere Noten. Trotz dieser Probleme kifft Kaja weiter. | Kaja is 16 years old, still in school, and smokes weed daily. The consumption is so intense that Kaja has developed a cannabis addiction. As soon as the day begins, Kaja thinks about smoking weed and often can't think about anything else. All of Kaja's friends also smoke weed and support Kaja's habit. Kaja has tried several times to reduce consumption, but it has never worked. Due to smoking weed, Kaja has had problems with concentration, attends school less and less, and gets increasingly worse grades. Despite these problems, Kaja continues to smoke weed. |
| Conny ist 50 Jahre alt, arbeitet in einem Getränkemarkt und kifft täglich. Der Konsum ist so ausgeprägt, dass sich bei Conny eine Cannabisabhängigkeit entwickelt hat. Sobald der Tag beginnt, denkt Conny ans Kiffen und kann oft an nichts anderes denken. Die meisten Menschen in Connys Umfeld kiffen auch und unterstützen den Konsum. Conny hat schon mehrmals probiert, den Konsum zu reduzieren, aber das hat nie geklappt. Durch das Kiffen hat Conny Probleme mit der Konzentration bekommen und geht auch immer seltener zur Arbeit bzw. kann den Aufgaben auf der Arbeit oft nicht nachkommen. Trotz dieser Probleme kifft Conny weiter. | Conny is 50 years old, works in a beverage store, and smokes weed daily. The consumption is so intense that Conny has developed a cannabis addiction. As soon as the day begins, Conny thinks about smoking weed and often can't think about anything else. Most of the people in Conny's social circle also smoke weed and support the habit. Conny has tried several times to reduce consumption, but it has never worked. Due to smoking weed, Conny has had problems with concentration, goes to work less frequently, and often cannot fulfill tasks at work. Despite these problems, Conny continues to smoke weed. |

*Supplemental Table 2.* Items of the scale related to general stigmatizing attitudes.

| **Item number** | **Original German item** | **English translation** |
| --- | --- | --- |
| 1 | Cannabisabhängigkeit entsteht aus einer Charakterschwäche | Cannabis dependence is the result of a weakness in character |
| 2 | Menschen mit einer Cannabisabhängigkeit sind selbst schuld an ihrer Situation | People with a cannabis dependence are at fault for being in this situation |
| 3 | Mit mehr Disziplin könnten Menschen mit Cannabisabhängigkeit ihren Konsum kontrollieren | People with cannabis dependence could control their cannabis use with more discipline |
| 4 | Ein Mensch mit einer Cannabisabhängigkeit kann nicht ohne therapeutische Hilfe aufhören, zu kiffen | A person with cannabis dependence cannot stop smoking cannabis without therapeutic support |
| 5 | Eine Cannabisabhängigkeit ist fast immer das Resultat von psychologischen Problemen | A cannabis dependence is almost always the result of psychological problems |
| 6 | Cannabisabhängigkeit ist eine Krankheit wie jede andere auch | Cannabis dependence is a disease like any other |
| 7 | Cannabis zu Rauschzwecken zu konsumieren ist eine akzeptable Lebensgewohnheit | Using cannabis to get high is an acceptable lifestyle |

**Supplemental results section**

*Supplemental Table 3.* Mean values (and SD) of the attribution questionnaire total score for each condition and each RM-ANOVA.

|  | Yes/Male | | | | No/Female | | | |
| --- | --- | --- | --- | --- | --- | --- | --- | --- |
|  | Adolescent participants | | Adult participants | | Adolescent participants | | Adult participants | |
|  | *Adolescent case* | *Adult case* | *Adolescent case* | *Adult case* | *Adolescent case* | *Adult case* | *Adolescent case* | *Adult case* |
| Did you ever use cannabis? | 32.34 (7.4) | 30.88 (7.9) | 34.70 (10.1) | 32.77 (10.7) | 36.62 (7.5) | 34.79 (8.0) | 38.22 (9.3) | 36.83 (10.4) |
| Did you use cannabis in the last 3 months? | 29.56 (6.5) | 28.34 (6.9) | 35.35 (12.2) | 33.82 (12.6) | 36.13 (7.5) | 34.35 (8.0) | 36.79 (9.5) | 35.16 (10.5) |
| Did you experience craving for cannabis use in the last 3 months? | 33.84 (7.3) | 32.20 (8.2) | 37.87 (12.9) | 36.65 (13.8) | 35.33 (7.7) | 33.62 (8.2) | 36.56 (9.6) | 34.91 (10.5) |
| In the last 3 months, was someone worried about your cannabis use? | 33.54 (8.4) | 32.36 (8.0) | 41.34 (12.2) | 41.41 (12.6) | 35.30 (7.6) | 33.56 (8.2) | 36.53 (9.6) | 34.62 (10.5) |
| Do you know someone with a cannabis use disorder? | 33.92 (8.0) | 32.04 (8.4) | 36.25 (10.0) | 34.59 (10.8) | 36.20 (7.3) | 34.62 (7.8) | 36.83 (9.8) | 35.23 (10.7) |
| Gender | 35.46 (7.7) | 33.70 (8.4) | 36.82 (10.3) | 35.26 (11.3) | 34.93 (7.7) | 33.29 (8.0) | 36.47 (9.34) | 34.77 (10.1) |

*Supplemental Table 4.* The effects of all included factors and their interactions across the five conduced RM-ANCOVAS.

|  | **Lifetime cannabis use** | **Cannabis use past 3 months** | **Craving past 3 months** | **Someone worried about use past 3 months** | **Know someone with cannabis use disorder** | **Gender** |
| --- | --- | --- | --- | --- | --- | --- |
| **Adolescent vs. adult vignette** | F(1,3196) = 12.77; *p* < .001 | F(1,3196) = 12.48; *p* < .001 | F(1,3196) = 12.28; *p* < .001 | F(1,3196) = 12.44; *p* < .001 | F(1,3196) = 12.34; *p* < .001 | F(1,3196) = 12.27; *p* < .001 |
| **Adolescent vs. adult participant** | F(1,3196) = 0.01; *p* = .94 | F(1,3196) = 0.01; *p* = .94 | F(1,3196) = 0.01; *p* = .94 | F(1,3196) = 0.01; *p* = .94 | F(1,3196) = 0.01; *p* = .94 | F(1,3196) = 0.006; *p* = .94 |
| **Cannabis use question/Gender** | F(1,3196) = 132.45; *p* < .001 | F(1,3196) = 37.56; *p* < .001 | F(1,3196) = 0.18; *p* = .67 | F(1,3196) = 23.87; *p* < .001 | F(1,3196) = 13.23; *p* < .001 | F(1,3196) = 1.31; *p* = .25 |
| **Vignette*participant** | F(1,3196) = 0.06; *p* = .81 | F(1,3196) = 0.05; *p* = .82 | F(1,3196) = 0.05; *p* = .82 | F(1,3196) = 0.05; *p* = .82 | F(1,3196) = 0.05; *p* = .82 | F(1,3196) = 0.053; *p* = .81 |
| **Vignette*cannabis/gender** | F(1,3196) = 0.18; *p* = .67 | F(1,3196) = 0.06; *p* = .81 | F(1,3196) = 0.04; *p* = .84 | F(1,3196) = 0.92; *p* = .33 | F(1,3196) = 0.05; *p* = .82 | F(1,3196) = 0.004; *p* = .94 |
| **Participant*cannabis/gender** | F(1,3196) = 0.03; *p* = .86 | F(1,3196) = 19.65; *p* < .001 | F(1,3196) = 4.89; *p =* .027 | F(1,3196) = 21.67; *p* < .001 | F(1,3196) = 6.00; *p =* .014 | F(1,3196) = 0.018; *p* = .89 |
| **Vignette*participant*cannabis/gender** | F(1,3196) = 0.41; *p* = .52 | F(1,3196) = 0.04; *p* = .84 | F(1,3196) = 0.02; *p* = .89 | F(1,3196) = 0.15; *p* = .69 | F(1,3196) = 0.03; *p* = .87 | F(1,3196) = 0.028; *p* = .87 |
